# An Endoscope-like SERS Probe Based on the Focusing Effect of Silica Nanospheres for Tyrosine and Urea Detection in Sweat

**DOI:** 10.3390/nano12030421

**Published:** 2022-01-27

**Authors:** Rongyuan Cai, Lijun Yin, Qian Huang, Ruiyun You, Shangyuan Feng, Yudong Lu

**Affiliations:** 1 Engineering Research Center of Industrial Biocatalysis, Fujian Provincial Key Laboratory of Advanced Oriented Chemical Engineer, Fujian Province Higher Education Institutes, College of Chemistry and Materials Science, Fujian Normal University, Fuzhou 350007, China; ccairongyuan@163.com (R.C.); yyinlj666@163.com (L.Y.); HuangQianfanfan@163.com (Q.H.); 2 Shaanxi Science and Technology Exchange Center, Xi’an 710054, China; 3 Key Laboratory of Optoelectronic Science and Technology for Medicine of Ministry of Education, Fujian Provincial Key Laboratory of Photonics Technology, Fujian Normal University, Fuzhou 350007, China; syfeng@fjnu.edu.cn

**Keywords:** SERS, endoscope, internal standard, tyrosine, urea, sweat

## Abstract

In this work, we developed a new type of SERS probe, which was composed of glass-SiO_2_-Au@MBN@Ag nanoparticles (NPs) three-dimensional Surface-enhanced Raman spectroscopy (SERS) substrate. When the laser passed through the quartz glass sheet, on the one hand, the SiO_2_ NPs supporting the Au@MBN@Ag NPs increase the roughness of the substrate surface, resulting in a large number of hot spots among nanoparticles. On the other hand, based on the focusing effect of silicon dioxide nanospheres, the laser can better focus on the surface of nanoparticles in the inverted SERS probe, thus showing better SERS enhancement. Furthermore, the Au@MBN@Ag NPs core-shell structure was used with 4-mercaptobenzoonitrile (MBN) as an internal standard molecule, and the quantitative determination of tyrosine and urea was realized by internal standard correction method. The standard working curves of the two had good linear correlation with R^2^ above 0.9555. The detection limits of tyrosine and urea were in the range of 2.85 × 10^−10^ M~7.54 × 10^−6^ M, which confirms that this design can be used for quantitative and specific detection of biological molecules, demonstrating great practical significance for the research of diseases such as skin lesions and endocrine disorders.

## 1. Introduction

Tyrosine and urea are important biomass in the human body [1]. Many metabolic disorders are associated with abnormal levels of these in body fluids, such as tyrosinemia [2], liver disease [3], neuropsychiatric disorders [4], and end-stage renal disease (ESRD) [5]. Recently, many studies on the detection of tyrosine and urea content in serum and urine have been proposed. However, there are still few studies on detecting these two kinds of content in sweat [6]. Moreover, on account of the low concentration of tyrosine in sweat, the measurement of tyrosine content in sweat is challenging.

Researchers have developed a variety of tyrosine detection methods, such as fluorescence spectroscopy [7], high performance liquid chromatography [8], electrochemical detection [9], colorimetry [10], and chemiluminescence [11]. However, these detection methods often have many disadvantages such as time-consuming detection and complex pretreatment process. The commonly used methods for urea detection include the electrochemical sensing method [12], flow injection method [13], ion chromatography [14], high performance liquid chromatography [15], and fluorescence spectroscopy [16], among others. These traditional methods are usually expensive and require complex instruments, as well as time-consuming and complex sample pretreatment process, making them unsuitable for field monitoring. Therefore, it is urgent to develop a rapid, non-invasive, and simple detection method of tyrosine and urea in sweat.

SERS has been widely used in various fields as an analytical technique with high sensitivity [17], high selectivity, and rapid diagnosis of diseases [18]. Many researchers have used SERS technology to detect tyrosine and urea. For example, Min-Liang Cheng et al. prepared a highly sensitive SERS substrate based on the deposition of silver nanoparticles on commercially available filter paper for selective detection of tyrosine in aqueous solution [19]. The linear range is up to 100 μM and the detection limit is 625 nM. Zhansaya Mukanova et al. deposited gold nanoparticles on aluminum foil for the detection of urea in human urine, showing good linearity in the range of 0.03~0.15 M; the detection limit was 0.017 M [20]. Although sensitive and promising, these works still suffer from some limitations. Raman signals are easily affected by fingerprints of biomolecular region (300–1800 cm^−1^) [21], and colloidal nanoparticles are prone to agglomeration and instability [22]. We found that self-assembly of relatively large Ag NPs on SiO_2_ nanospheres not only increased the surface roughness of the substrate, but also produced a large number of hot spots between Ag NPs [23]. Therefore, the glass-SiO_2_-Ag NPs SERS substrate can achieve better SERS intensity and good reusability compared to glass-Ag NPs SERS substrate. Furthermore, compared with the test results of direct laser irradiation on the surface of the 3D SERS substrate, the unexpected SERS enhancement can be obtained by changing the detection method.

**Scheme 1:**
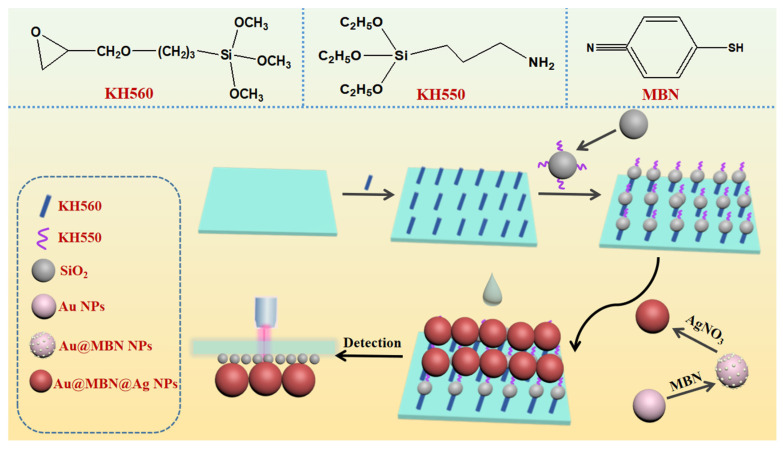
Schematic of the synthesis of glass-SiO_2_-Au@MBN@Ag SERS probe.

On the basis of the above research, we designed a further kind of glass-SiO_2_-Au@MBN@Ag NPs SERS probe in this work. All surface functionalization only occurs on one side of the glass plate. As shown in Scheme 1, smaller SiO_2_ nanospheres was self-assembled on glass to form a monolayer and then relatively large Au@MBN@Ag NPs were deposited on the SiO_2_ nanospheres to constitute the SERS probe. The prepared SERS probe was inverted, using the focusing effect of silica nanospheres, and the laser beam was focused through the glass to Au@MBN@Ag NPs surface on the back side to detect tyrosine and urea, which formed an endoscope-like SERS probe. At the same time, the Au@MBN@Ag NPs core-shell structure was used. A unique internal standard molecule, MBN, has a unique characteristic peak at 2228 cm^−1^ corresponding to the ν (C≡N) stretch mode, which can avoid the interference of molecular bio-regional signals (<1800 cm^−1^) [24]. Therefore, with the help of MBN as an internal standard molecule, the quantitative determination of tyrosine and urea was realized by the internal standard calibration method.

## 2. Experimental Section

### 2.1. Chemicals and Materials

Chloro-auric acid tetrahydrate was purchased from Shenbo Chemical Co., Ltd. (Shenzhen, China) Ascorbic acid (AA), trisodium citrate, silver nitrate, sodium carbonate, and 4-aminothiophenol (p-ATP) were purchased from Aladdin (Shanghai, China) Co., Ltd. and directly used without other treatments. 4-mercaptobenzoonitrile (MBN) was purchased from Shanghai Haohong Biomedical Technology Co., Ltd. (Shanghai, China) Glacial acetic acid, concentrated sulfuric acid, and hydrogen peroxide were purchased from Sinopharm Chemical Reagent Co., Ltd. (Shanghai, China), and were directly used without other treatments. The glass slides were purchased from SAIL Brand Co., Ltd. (Suzhou, China), 0.5 mm/0.25 mm quartz glass sheets were purchased from Donghai Ailfa Quartz Products Co., Ltd. 3-aminopropyltriethoxysilane (KH550) and γ-(2,3-epoxypropoxy) propytrimethoxysilane (KH560) were purchased from Guangzhou Jucheng Zhaoye Organic Silicon Raw Material Co., Ltd. (Guangzhou, China), SiO_2_ nanoparticles were purchased from Hunyuan Junhong New Materials Co., Ltd. Urea and anhydrous ethanol were purchased from Xilong Technology Co., Ltd. Tyrosine, tryptophan, serine, threonine, histidine, cysteine, and lysine were purchased from Shanghai Sangon Bioengineering Co., Ltd., (Shanghai, China).

### 2.2. Instrumentation

UV–Vis spectra were obtained using a Lambda 950 (PerkinElmer, Waltham, MA, USA). Scanning electron microscopy (SEM) images were obtained using a SU 8010 (Hitachi, Tokyo, Japan). Transmission electron microscopy (TEM) images were obtained using a Tecnai-G20 (FEI, Hillsboro, OR, USA). All SERS spectra of sample were recorded with a confocal Raman microspectrometer (Renishaw, London, UK), which was equipped with a 785nm diode laser (4.9 mW) and a Peltier cooled charge coupled device camera (spectral resolution of 2 cm^−1^) for spectral detection in the range of 400~2500 cm^−1^. Before measurement, the Raman spectrometer was calibrated using the standard Raman spectrum of silicon, whose first order Raman peak was centered at 520 cm^−1^. The SERS spectrum was collected from three random points on each of solid substrate.

### 2.3. Synthesis of Au@MBN@Ag NPs

First, 100 mL 0.01% HAuCl_4_ solution was heated and stirred until it boiled, then 2 mL of 1% trisodium citrate aqueous solution was added, and the color of the solution changed from light yellow to red, continue heating and stirring for 15 min to form spherical Au NPs. Finally, the gold nanoparticles were cooled to room temperature for later use.

10 mL Au NPs was added to 50 µL MBN (10^−4^ M), stirred for 2 h, centrifugally washed once, and resuspended to 10 mL. 10 mL Au NPs solution containing internal standard was taken, and 1 mL 1 mM AgNO_3_ solution was slowly added. After stirring for 2 min, 250 μL AA (0.01 M) was added drop by drop. After stirring for 30 min, the solution was suspended by centrifugation with distilled water for later use.

### 2.4. Modification of Glass Slides

First, prepare 0.5 cm × 0.5 cm glass slices and ultrasonically clean them in anhydrous ethanol for 15 min. After drying, they were soaked in piranha solution and placed in a 90 °C oven for 120 min. Subsequently, the functionalized glass slides were washed three times with water and ethanol, respectively, and dried for later use. A 20% (2,3-epoxy propoxy) propyltrimethoxysilane solution (*w*/*w*) (containing 20% silane coupling agent, 72% methanol, 4% water, 4% glacial acetic acid) was prepared. The glass slices were immersed in 20% (2,3-epoxy propoxy) propyltrimethoxysilane for 24 h, cleaned with ethanol for 3 times, and placed at 120 °C for 15 min.

### 2.5. Modification of SiO_2_ Nanospheres

10 mL 1% SiO_2_ ethanol solution (ethanol:water = 4:1) and 1 mL 3-trimethoxypropylamine solution were mixed and stirred for 24 h, centrifuged and cleaned with ethanol for 3 times, dried at 50 °C, and then redispersed in the ethanol solution (ethanol:water = 4:1).

### 2.6. Preparation of Glass-SiO_2_-Au@MBN@Ag NPs SERS Probe

The modified glass pieces were soaked in the modified silica solution for 24 h. After cleaning and drying, 6 µL Au@MBN@Ag NPs, which was 10 times concentrated, was dropped on one side of the glass to make the Au@MBN@Ag NPs self-assemble on glass-SiO_2_.

### 2.7. Detection of Tyrosine and Urea

Detection of tyrosine: reagents used in diazo coupling reactions were prepared according to previously reported methods. Firstly, different concentrations of tyrosine solutions were prepared, and then appropriate amount of 4-aminothiophenol (p-ATP) was dissolved in 0.5 mL HCl (12 M) and diluted to 50 mL with distilled water to obtain reagent A1 (10^−3^ M p-ATP solution); reagent A2 was prepared by dissolving NaNO_2_ in distilled water (5%, *w*/*v*). Reagent B was prepared by dissolving Na_2_CO_3_ in distilled water (8%, *w*/*v*). All of these reagents were stored at 4 °C prior to use. In the diazo coupling reaction, the mixture ratio of reagent A1:reagent A2:reagent B:Tyrosine is 1:1:1:2 (*v*/*v*/*v*/*v*). The specific operation steps were as follows: first, reagent A2 was added to reagent A1, and the reagent was stirred mechanically in an ice water bath for 1 min. Then, the tyrosine sample and reagent B were quickly added to the above solution and stirred for 1 min. When the coupling reaction had completed, the obtained 1.5 μL mixture was immediately dropped on the modified side of the nanoparticles in the glass-SiO_2_-Au@MBN@Ag NPs SERS probe. Finally, after the object to be measured was evaporated and dried, the glass-SiO_2_-Au@MBN@Ag NPs SERS probe was reversed to conduct SERS test and collect its Raman signal.

Detection of urea: first, different concentrations of urea solutions were prepared, and appropriate amount of urea solution was dropped on one side of the modified nanoparticles in the glass-SiO_2_-Au@MBN@Ag NPs SERS probe. Finally, after evaporation and drying of the objects to be measured, the glass-SiO_2_-Au@MBN@Ag NPs SERS probe was reversed to conduct SERS test and collect its Raman signal.

### 2.8. Detection of Tyrosine and Urea in Skin Sweat

The sweat produced was collected and placed after moderate exercise in a beaker for ultrasound for 10 min, and then centrifuged at 10,000 r/min for 10 min. The supernatant was taken and filtered with 0.22 μm nylon filter head.

We prepared a series of concentrations urea (50 μM, 100 μM, 500 μM) and tyrosine (50 μM, 100 μM, 200 μM) in sweat, and the spiked samples were individually analyzed using our glass-SiO_2_-Au@MBN@Ag NPs SERS probe. Finally, the SERS recovery rates of tyrosine and urea in sweat were calculated using the equations obtained from linear analysis.

## 3. Results and Discussion

### 3.1. Design Mechanism

**Figure 1:**
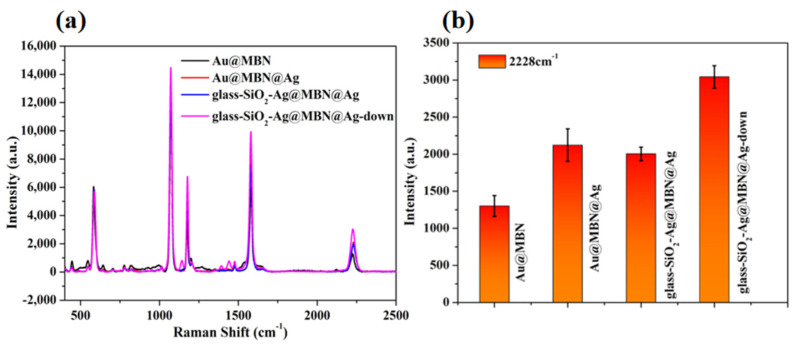
(**a**) SERS spectra of Au@MBN NPs, Au@MBN@Ag NPs, glass-SiO_2_-Au@MBN@Ag NPs SERS substrate and glass-SiO_2_-Au@MBN@Ag NPs SERS probe; (**b**) SERS signal intensity at 2228 cm^−1^ of Au@MBN NPs, Au@MBN@Ag NPs, glass-SiO_2_-Au@MBN@Ag NPs SERS substrate and glass-SiO_2_-Au@MBN@Ag NPs SERS probe.

In order to verify the application potential of SERS probe in this work, we compared the SERS intensities of four different sets of probes. The measured SERS spectra are shown in Figure 1a, which correspond to Au@MBN NPs (black), Au@MBN@Ag NPs (red), glass-SiO_2_-Au@MBN@Ag NPs SERS substrate (blue) and our SERS probe in this work (purple). When comparing the SERS intensities of Au@MBN@Ag NPs and glass-SiO_2_-Au@MBN@Ag NPs SERS substrate at the characteristic peak at 2228 cm^−1^, it was found that there was no significant difference between the two. However, when the laser passed through the quartz glass sheet, due to the focusing effect of silica nanospheres, the light was focused on the Au@MBN@Ag NPs, making their SERS signal significantly enhanced (Figure 1b). We call this arrangement the “down” setup.

**Figure 2:**
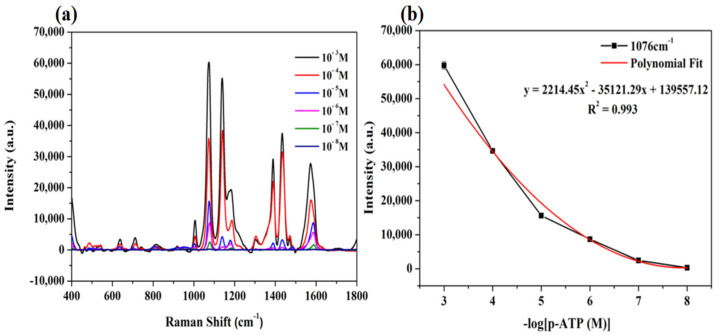
(**a**) SERS spectra of different concentrations of p-ATP; (**b**) Plot of SERS signal intensities at 1076 cm^−1^ versus p-ATP concentration.

In order to explore the feasibility of this method, further experiments were carried out on the SERS substrate. Ten μL of different concentrations of p-ATP solution were added to the side containing silver nanoparticles, and the SERS signal was measured in the “down” setup. The SERS spectra are shown in Figure 2a, when plotting the absolute Raman intensity of different concentrations of p-ATP at the characteristic peak at 1076 cm^−1^, a good correlation can be obtained, and the polynomial fit value R^2^ was 0.993 (Figure 2b).

### 3.2. Characterization of Au@MBN@Ag NPs

**Figure 3:**
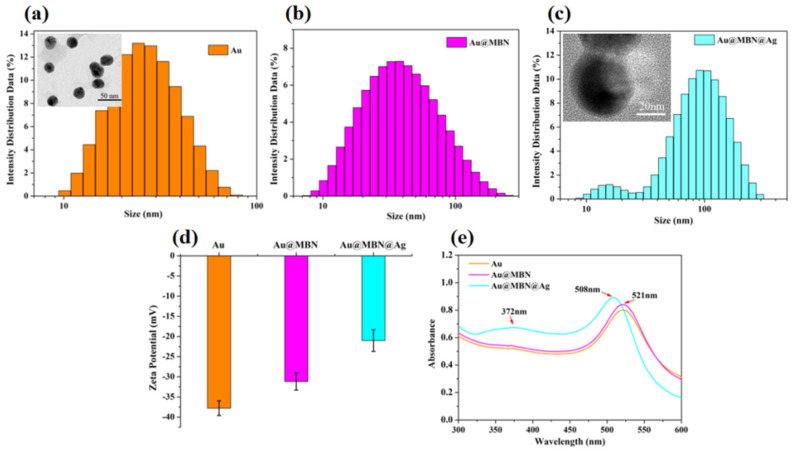
(**a**) Particle size distribution of Au NPs (the illustration is the TEM image of the Au NPs); (**b**) Au@MBN NPs particle size distribution; (**c**) Au@MBN@Ag NPs particle size distribution (the illustration is the TEM image of the Au@MBN@Ag NPs); Zeta potential (**d**) and UV-Vis absorption spectra (**e**) of Au NPs, Au@MBN NPs, and Au@MBN@Ag NPs.

The uniform morphology and good properties of Au@MBN@Ag NPs were prepared by the experimental method in this work. First, uniform quasi spherical Au NPs were prepared by sodium citrate reduction method, then internal standard molecules were added for incubation so that the nanoparticles were wrapped around by internal standard molecules, and then appropriate amount of ascorbic acid and silver nitrate was added to make the outer layer of the dense silver shell grow. The particle size of the prepared nanoparticles was tested, and its particle size distribution was shown in Figure 3a–c. As the preparation steps went on, the measured particle size gradually increased. Zeta potential test was carried out on the prepared nanoparticles. As shown in Figure 3d, with the inclusion of internal standard molecules and the growth of silver shell layer, the Zeta potential gradually became positive. In Figure 3e, the UV absorption spectra of Au NPs, Au@MBN and Au@MBN@Ag NPs are shown. It can be clearly found that the silver shell layer adds a characteristic peak at 372 nm. Meanwhile, the characteristic peak representing the gold core was also blue shifted from 521 nm to 508 nm.

### 3.3. Characterization of Supported SiO_2_

**Figure 4:**
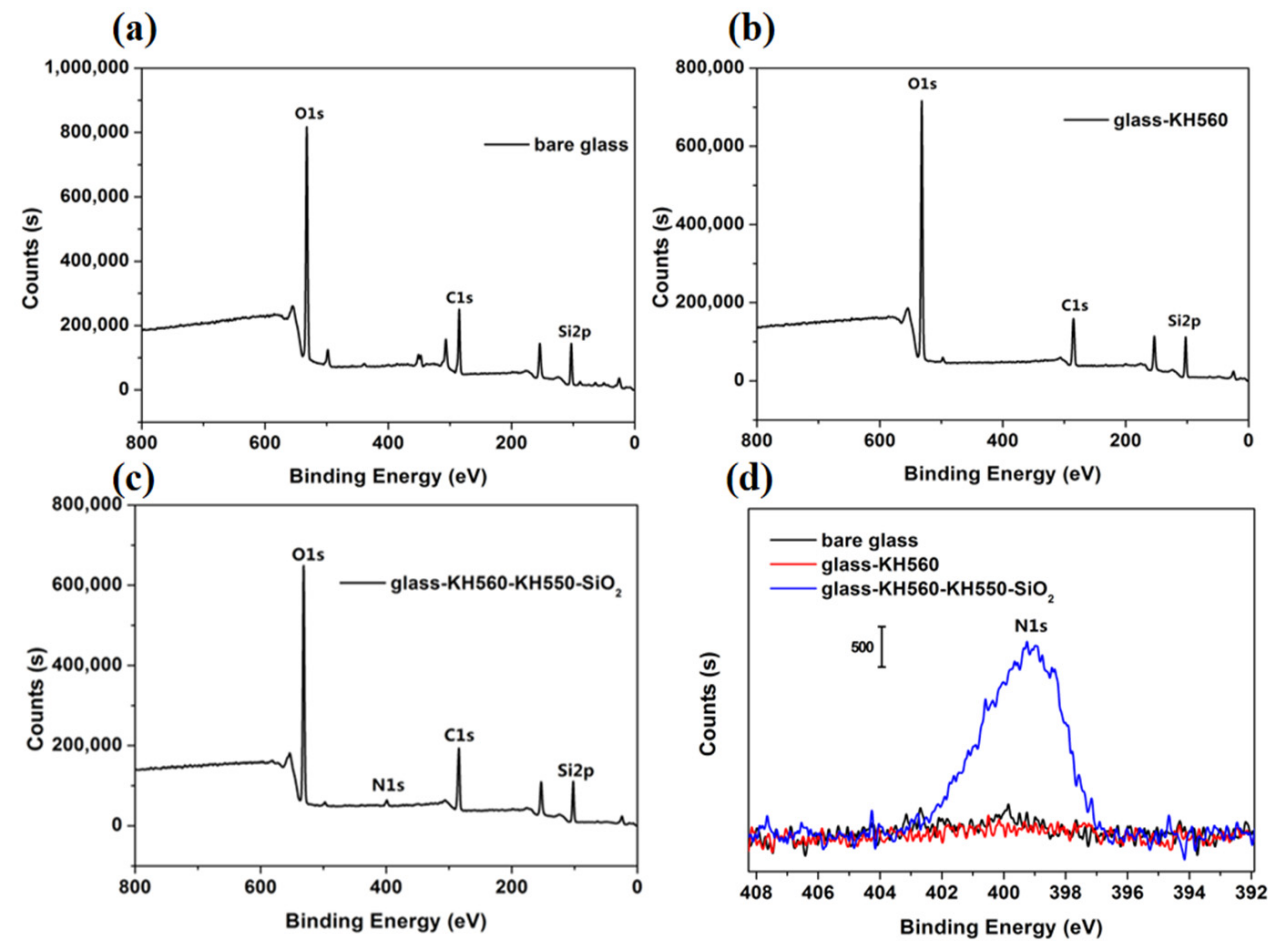
XPS spectra of (**a**) Bare glass; (**b**) Glass-KH560; (**c**) Glass-KH560- KH550-SiO_2_; (**d**) XPS analysis spectrum of N element.

In order to verify the successful loading of SiO_2_ on the glass sheet, XPS of bare glass sheet, glass sheet loaded with KH560 and glass sheet loaded with silica by the method presented in this work were tested respectively. The measured XPS images are shown in Figure 4, the results show that only the glass sheet loaded with silica contains N element, corresponding to the amino group on KH550. It shows that SiO_2_ had been successfully loaded on the glass sheet through the reaction between KH550 and KH560. 

### 3.4. Conditions Optimization

**Figure 5:**
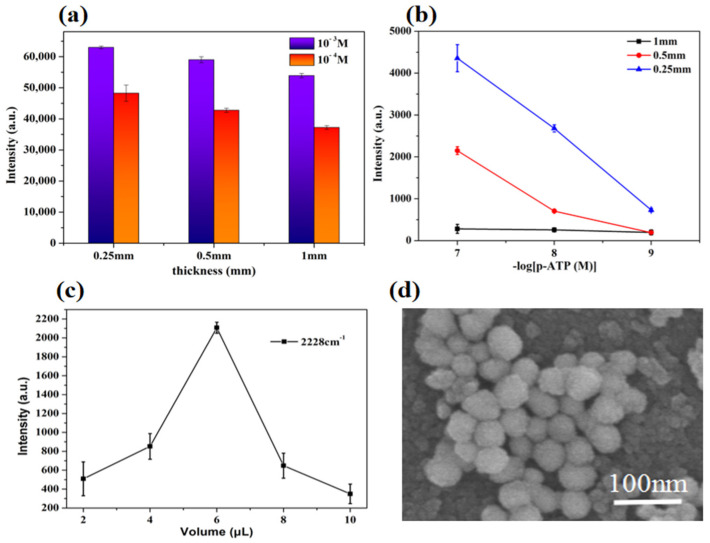
The intensity distribution of 1076 cm^−1^ characteristic peak in SERS spectra detected by glass slices of different thicknesses with different concentrations of p-ATP; (**a**) Signal intensity comparison at high concentration; (**b**) Comparison of detection limits at low concentrations; (**c**) The intensity line chart of 2228 cm^−1^ characteristic peak of different volumes Au@MBN@Au NPs were added; (**d**) SEM image of glass-SiO_2_-Au@MBN@Ag NPs SERS probe.

During the detection process, the thickness of the glass sheet causes a loss in the laser power. Therefore, the glass sheet thickness was optimized to obtain the highest possible SERS enhancement. In this experiment, three kinds of quartz glass sheets with different thicknesses were selected to prepare SERS probes (0.25 mm, 0.5 mm and 1 mm), and different concentrations of p-ATP were detected. As shown in Figure 5a,b, the SERS intensity of the characteristic peak of p-ATP at 1076 cm^−1^ increased with the decrease in the thickness of quartz glass sheets at high concentration. At low concentrations (10^−7^ M~10^−9^ M), SERS probes with thicknesses of 1 mm and 0.5 mm could hardly detect the SERS signal. While the SERS probe with a thickness of 0.25 mm can clearly detect the SERS characteristic peak of p-ATP even at the concentration of 10^−9^ M. Therefore, 0.25 mm quartz glass sheet was selected as the optimal thickness for subsequent experiments.

The amount of nanoparticles loaded on the glass sheet was optimized. The SERS probes modified with different amounts of Au@MBN@Ag NPs were used for SERS tests. As shown in Figure 5c, 2~6 μL of Au@MBN@Ag NPs was modified, and the characteristic peak intensity of the internal standard molecule MBN at 2228 cm^−1^ increased with the increase of nanoparticles concentration and reached the best at 6 μL. On the contrary, when the nanoparticle concentration was greater than 6 μL, the intensity of the characteristic peak at 2228 cm^−1^ gradually weakened. This was due to the increase in the thickness of the nanoparticles, and the laser needs to pass through a thicker layer of silver nanoparticles, resulting in laser loss. The glass-SiO_2_-Au@MBN@Ag NPs SERS probe was prepared under the above optimal conditions. Its SEM image shows that the Au@MBN@Ag NPs were uniformly loaded on the SiO_2_ nanospheres (Figure 5d).

### 3.5. Anti-Interference Experiment

**Scheme 2:**
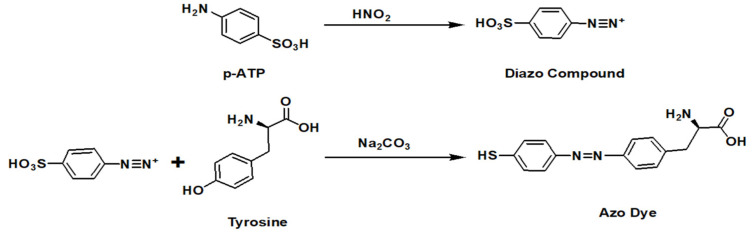
Diazo reaction principle of tyrosine.

Amino acids and urea are involved in various metabolic cycles in the human body, so their specific detection is very important. Fortunately, urea has a unique and sharp SERS characteristic peak at 1003 cm^−1^, corresponding to the -N-C-N- stretching vibration mode, which can be directly detected. In contrast, tyrosine is very low in human metabolites (sweat) and its normal Raman scattering is weak, so it is not easy to detect. In view of the above challenges, we adopted a diazo coupling reaction to achieve the specific detection of tyrosine. The reaction principle is shown in Scheme 2, p-ATP is oxidized with HNO_2_ under low temperature and acidic conditions to form diazo compounds, which can obtain azo compounds by nucleophilic attack with tyrosine. This azo compound produces unique and strong SERS signals at 1139 cm^−1^, 1388 cm^−1^, and 1436 cm^−1^, respectively [25].

**Figure 6:**
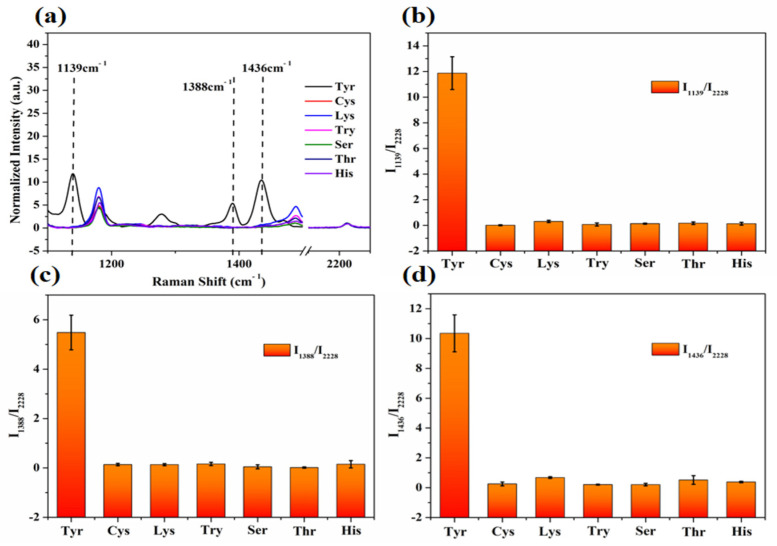
(**a**) Normalized SERS spectra of tyrosine, lysine, threonine, histidine, tryptophan, cysteine, and serine detected by diazo reaction; (**b**) Histogram of the ratio of the normalized intensity of characteristic peaks 1139 cm^−1^ and 2228 cm^−1^ in SERS spectra of tyrosine and lysine, threonine, histidine, tryptophan, cysteine, and serine; (**c**) Histogram of the ratio of the normalized intensity of characteristic peaks 1388 cm^−1^ and 2228 cm^−1^ in SERS spectra of tyrosine and lysine, threonine, histidine, tryptophan, cysteine, and serine; (**d**) Histogram of the ratio of the normalized intensity of characteristic peaks 1436 cm^−1^ and 2228 cm^−1^ in SERS spectra of tyrosine and lysine, threonine, histidine, tryptophan, cysteine, and serine.

Metabolites in sweat are a complex environment that contains a variety of amino acids. Therefore, we selected several common amino acids as interferors for identification, such as lysine, threonine, histidine, tryptophan, cysteine, and serine. When these interfering substances were added into the diazo reaction process instead of tyrosine, it was found that only in the presence of tyrosine, there were three distinct characteristic peaks of 1139 cm^−1^, 1388 cm^−1^, and 1436 cm^−1^ in SERS spectrum. The corresponding three characteristic peaks were not found in SERS spectra of other amino acids (Figure 6a). The ratio between the peak strength of the three characteristic peaks of tyrosine and the 2228 cm^−1^ characteristic peak was obviously different from that of other amino acids, indicating that the method had good specificity and other amino acids do not interfere with the detection of tyrosine (Figure 6b–d).

### 3.6. Detection of Tyrosine and Urea

**Figure 7:**
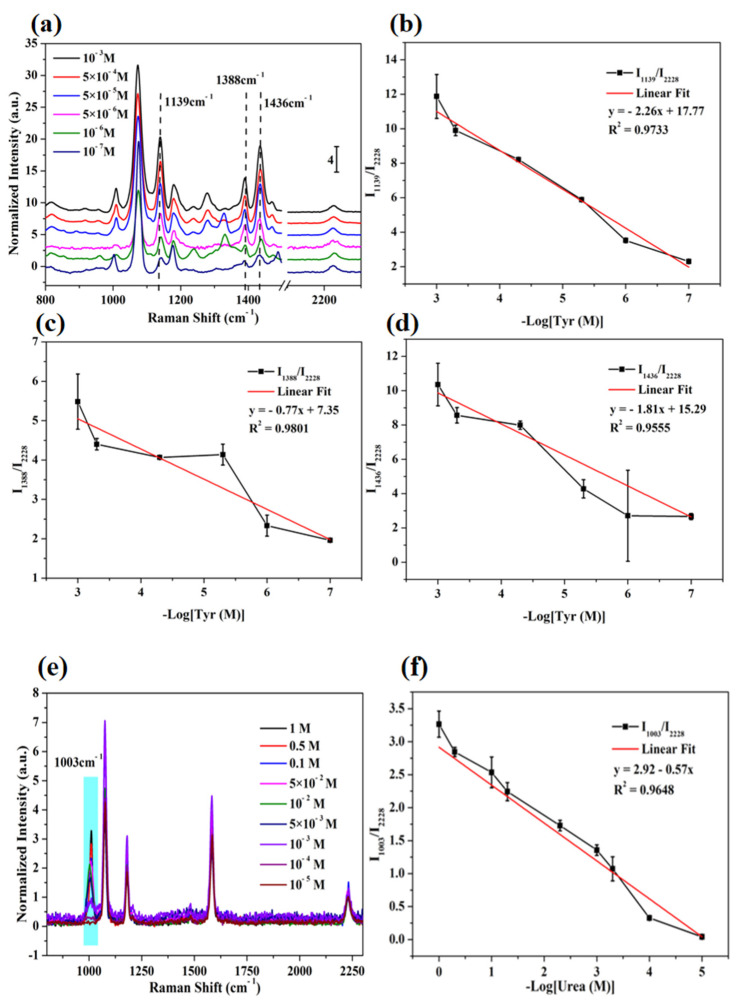
(**a**) SERS spectra of different concentrations of tyrosine; (**b**) linear analysis of the ratio of the normalized intensity of 1139 cm^−1^ characteristic peak to the normalized intensity of 2228 cm^−1^ characteristic peak and the logarithm of tyrosine concentration in SERS spectrum; (**c**) linear analysis of the ratio of the normalized intensity of 1388 cm^−1^ characteristic peak to the normalized intensity of 2228 cm^−1^ characteristic peak and the logarithm of tyrosine concentration in SERS spectrum; (**d**) linear analysis of the ratio of the normalized intensity of 1436 cm^−1^ characteristic peak to the normalized intensity of 2228 cm^−1^ characteristic peak and the logarithm of tyrosine concentration in SERS spectrum; (**e**) SERS spectra of different concentrations of urea; (**f**) linear analysis of the ratio of the normalized intensity of 1003 cm^−1^ characteristic peak to the normalized intensity of 2228 cm^−1^ characteristic peak and the logarithm of urea concentration in SERS spectrum.

After above optimization experiments and specific experiments, we used glass-SiO_2_-Au@MBN@Ag NPs SERS probe to construct the standard working curves of tyrosine and urea. The corresponding SERS spectra of different concentrations of tyrosine after diazo are shown in Figure 7a. It can be seen that the tyrosine concentration was positively correlated with the characteristic peak corresponding to the azo product. The standard working curves of tyrosine from 10^−3^ M to 10^−7^ M are shown in Figure 7a, after intensity normalization, the intensity of characteristic peak at 2228 cm^−1^ in the silent zone of MBN is certain. Meanwhile, the peak strength of characteristic peaks at 1139 cm^−1^, 1388 cm^−1^, and 1436 cm^−1^ increased with the increase in tyrosine concentration. As shown in Figure 7b–d, within the range of 10^−3^ M~10^−7^ M, the ratio of SERS intensity of characteristic peaks at 1139 cm^−1^, 1388 cm^−1^, 1436 cm^−1^, and 2228 cm^−1^ had an ideal linear relationship with the negative number of logarithm of tyrosine concentration. The standard working curves’ expressions were y = −2.26 x + 17.77, y = −0.77 x + 7.35, y = −1.81 x + 15.29, and the linear regression values R^2^ was 0.9801. The detection limits were 1.37 × 10^−8^ M, 2.85 × 10^−10^ M and 3.57 × 10^−9^ M, respectively. Consequently, when the concentration of tyrosine was in the range of 0.1 μM to 1000 μM, the diazo reaction could be used for quantitative analysis of tyrosine.

SERS spectra of urea with different concentrations were detected and shown in Figure 7e. With the increase of urea concentration, the intensity of its SERS characteristic peak at 1003 cm^−1^ also increased, and at the same time, the ratio of its intensity to the calibrated peak at 2228 cm^−1^ also increased. Linear analysis was made of the ratio between the intensity of the two characteristic peaks and the logarithm of urea concentration. As shown in Figure 7f, they all showed an ideal linear relationship. The standard working curve was y = −0.57 x + 2.92, and the linear regression value R^2^ was 0.9648, the detection limit was 7.54 × 10^−6^ M.

The method proposed in this work was compared with other methods for the detection of tyrosine and urea in Table 1. Compared with our method, Wang et al. used the electrochemical method to detect urea with a detection limit of 5.88 µM, which was lower than the SERS probe designed by us. However, its detection concentration sensitivity range was far lower than that of this method. In addition, in real samples, tyrosine and urea were usually detected in blood or urine and rarely in sweat. In a word, compared with other methods, this method had the advantages of fast detection speed, low detection limit, and wide linear range.

### 3.7. Detection of Tyrosine and Urea in Real Samples

We added tyrosine to sweat and tested its recovery by diazo reaction. Different concentrations of tyrosine (C_i_) were added to the sweat samples for SERS detection. According to the linear equation (y = −2.26 x + 17.77, R^2^ = 0.9733, Figure 7b) of tyrosine standard solution, the real detected concentration of tyrosine in sweat (C_0_) was first calculated, and then the ratio of the two (C_0_/C_i_) was chosen as the recovery rate. The corresponding recoveries of different concentrations of tyrosine (50 μM, 100 μM, 200 μM) are shown in Table 2. It can be found that the SERS recovery rates of tyrosine detected in sweat were between 91.98% and 104.26%, and the three characteristic peaks all had very good recovery. This indicated that the method proposed in this work had great application potential in the detection of tyrosine in sweat during the detection of real samples.

Similarly, we added different concentrations of urea (50 μM, 100 μM, 500 μM) to sweat and obtained their corresponding recoveries. As shown in Table 3, the recovery rates of urea in sweat were 104.45%, 98.58%, and 109.03%, respectively. Similarly, the three different concentrations had very good recovery rates; the relative standard error (RSD) was between 2.65% and 7.75%, indicating that the method in this work also had great application potential in the detection of urea in sweat during the detection process of real samples.

## 4. Conclusions

In this work, a glass-SiO_2_-Au@MBN@Ag NPs SERS probe was designed to obtain excellent SERS enhancement by increasing the roughness and focusing effect of the substrate surface. Using diazo reaction to detect tyrosine not only had good specificity, but also good detection effect. The SERS intensity ratio of characteristic peaks corresponding to azo compounds and MBN had an ideal linear relationship with the negative logarithm of tyrosine concentration. The detection limits were 1.37 × 10^−8^ M, 2.85 × 10^−10^ M, and 3.57 × 10^−9^ M, respectively. Likewise, the SERS intensity ratio of urea (1003 cm^−1^) to MBN was ideally linear with the negative logarithm of the urea concentration. The content of tyrosine and urea in sweat was detected by using glass-SiO_2_-Au@MBN@Ag NPs SERS probe. When the tyrosine of 50 μM, 100 μM, and 200 μM were added to sweat, respectively, the SERS recovery was between 91.98~104.26%. Besides, the recoveries were 104.45%, 98.58%, and 109.03%, respectively, when urea of 50 μM, 100 μM, and 500 μM were added to sweat.

## Figures and Tables

**Table 1:** Comparison for determination of Tyr and urea using different methods.

Biomarkers	Methods	Application	Analytical Range	LOD	Reference
Tyr	Electrochemistry	Serum	30–500 µM	4.4 µM	[26]
	Colorimetry	Urine	5–100 µM	2.74 µM	[27]
	Fluorescence	Urine	5–100 µM	4.9 µM	[28]
	Light scattering probe	Biological samples	0.055–4.68 µM	0.0138 µM	[29]
	IR spectroscopic	Biological samples	10–200 µM	5.88 µM	[30]
	Our method	Sweat	0.1–10^3^ µM	0.00357 µM	This work
Urea	Electrochemistry	Aqueous solution and milk	10–200 µM	5.88 µM	[31]
	Enzymatic catalysis	Aqueous solution	1–30 mM	0.48 mM	[32]
	Electrochemistry	Alkaline buffer and tap water	0.05–2.5 mM	20 µM	[33]
	Electrochemistry	Human blood	1–8 mM	0.14 mM	[34]
	SERS	Aqueous solution	15–300 mM	0.1 mM	[35]
	Our method	Sweat	10–10^6^ µM	7.54 µM	This work

**Table 2:** SERS recovery test of spiked tyrosine in sweat.

Spiked (μM)	Characteristic Peak (cm^−1^)	SERS (μM)	SERS Recovery (%)	RSD (%)
200	1139	198.8	99.39	3.82
	1388	183.1	91.55	2.32
	1436	201.6	100.79	10.18
100	1139	96.6	96.58	1.11
	1388	99.9	99.92	1.54
	1436	96.7	96.72	2.17
50	1139	46.0	91.98	2.48
	1388	52.1	104.26	1.72
	1436	47.5	95.03	2.65

**Table 3:** SERS recovery test of spiked urea in sweat.

Spiked (μM)	Characteristic Peak (cm^−1^)	SERS (μM)	SERS Recovery (%)	RSD (%)
500	1003	545.1	109.03	7.75
100	1003	98.6	98.58	2.65
50	1003	52.2	104.45	3.69

## Data Availability

The data presented in this study are available on request from the corresponding author.

## References

[1] Celleno L. (2018). Topical urea in skincare: A review. Dermatol. Ther..

[2] Russo P.A., Mitchell G.A., Tanguay R.M. (2001). Tyrosinemia: A Review. Pediatric Dev. Pathol..

[3] Levine R.J., Conn H.O. (1967). Tyrosine Metabolism in Patients with Liver Disease. J. Clin. Investig..

[4] Capuron L., Schroecksnadel S., Féart C., Aubert A., Higueret D., Barberger-Gateau P., Layé S., Fuchs D. (2011). Chronic Low-Grade Inflammation in Elderly Persons Is Associated with Altered Tryptophan and Tyrosine Metabolism: Role in Neuropsychiatric Symptoms. Biol. Psychiatry.

[5] Keller R.W., Bailey J.L., Wang Y., Klein J.D., Sands J.M. (2016). Urea transporters and sweat response to uremia. Physiol. Rep..

[6] Liappis N., Kelderbacher S.D., Kesseler K., Bantzer P. (1979). Quantitative study of free amino acids in human eccrine sweat excreted from the forearms of healthy trained and untrained men during exercise. Eur. J. Appl. Physiol. Occup. Physiol..

[7] Wang P., An Y., Wu J. (2020). Highly sensitive turn-on detection of mercury(II) in aqueous solutions and live cells with a chemosensor based on tyrosine. Spectrochim. Acta Part A Mol. Biomol. Spectrosc..

[8] Neurauter G., Scholl-Bürgi S., Haara A., Geisler S., Mayersbach P., Schennach H., Fuchs D. (2013). Simultaneous measurement of phenylalanine and tyrosine by high performance liquid chromatography (HPLC) with fluorescence detection. Clin. Biochem..

[9] Deng P., Xiao J., Feng J., Tian Y., Wu Y., Li J., He Q. (2021). Highly sensitive electrochemical sensor for tyrosine detection using a sub-millimeter electrode. Microchem. J..

[10] Vyas G., Bhatt S., Si M.K., Jindani S., Suresh E., Ganguly B., Paul P. (2020). Colorimetric dual sensor for Cu(II) and tyrosine and its application as paper strips for detection in water and human saliva as real samples. Spectrochim. Acta Part A Mol. Biomol. Spectrosc..

[11] Pang C., Han S., Li Y., Zhang J. (2018). Graphene quantum dot-enhanced chemiluminescence through energy and electron transfer for the sensitive detection of tyrosine. J. Chin. Chem. Soc..

[12] Kyunghee K., Jeongeun L., Mi M.B., Been S.Y., Hum P.C., Min P., Yong S.G. (2018). Fabrication of a Urea Biosensor for Real-Time Dynamic Fluid Measurement. Sensors.

[13] Hu X., Takenaka N., Kitano M., Bandow H., Maeda Y., Hattori M. (1994). Determination of trace amounts of urea by using flow injection with chemiluminescence detection. Analyst.

[14] Miyauchi T., Miyachi Y., Takahashi M., Ishikawa N., Mori H. (2010). Determination of Urea in Serum Based on the Combination of an Enzymatic Reaction with Immobilized Urease and Ion Chromatographic Analysis. Anal. Sci..

[15] Krämer M., Fry H., Kappenstein O. (2021). Development and validation of two analytical methods for urea determination in compound feed, including pet food, and yeast using high-performance liquid chromatography coupled with fluorescence detection and tandem mass spectrometry. Food Addit. Contam. Part A.

[16] Soundharraj P., Dhinasekaran D., Rajendran A.R., Prakasarao A., Ganesan S. (2021). N-Doped zinc oxide as an effective fluorescence sensor for urea detection. New J. Chem..

[17] Wu Q., Chen G., Qiu S., Feng S., Lin D. (2021). A target-triggered and self-calibration aptasensor based on SERS for precise detection of a prostate cancer biomarker in human blood. Nanoscale.

[18] Zhang W.S., Wang Y.N., Xu Z.R. (2020). High sensitivity and non-background SERS detection of endogenous hydrogen sulfide in living cells using core-shell nanoparticles. Anal. Chim. Acta.

[19] Cheng M.-L., Tsai B.-C., Yang J. (2011). Silver nanoparticle-treated filter paper as a highly sensitive surface-enhanced Raman scattering (SERS) substrate for detection of tyrosine in aqueous solution. Anal. Chim. Acta.

[20] Mukanova Z., Gudun K., Elemessova Z., Khamkhash L., Ralchenko E., Bukasov R. (2018). Detection of Paracetamol in Water and Urea in Artificial Urine with Gold Nanoparticle@Al Foil Cost-efficient SERS Substrate. Anal. Sci..

[21] Lin X., Wang Y., Wang L., Lu Y., Li J., Lu D., Zhou T., Huang Z., Huang J., Huang H. (2019). Interference-free and high precision biosensor based on surface enhanced Raman spectroscopy integrated with surface molecularly imprinted polymer technology for tumor biomarker detection in human blood. Biosens. Bioelectron..

[22] Zhou Y., Li Y., Lu S., Ren H., Li Z., Zhang Y., Pan F., Liu W., Zhang J., Liu Z. (2010). Gold nanoparticle probe-based immunoassay as a new tool for tetrodotoxin detection in puffer fish tissues. Sens. Actuators B Chem..

[23] Cai R., Lu D., She Q., You R., Feng S., Lin X., Lu Y. (2021). Reusable 3D silver superposed silica SERS substrate based on the Griess reaction for the ratiometric detection of nitrite. Anal. Bioanal. Chem..

[24] Fan M., She Q., You R., Huang Y., Chen J., Su H., Lu Y. (2021). “On-off” SERS sensor triggered by IDO for non-interference and ultrasensitive quantitative detection of IDO. Sens. Actuators B Chem..

[25] Lu Y., Lu D., You R., Liu J., Huang L., Su J., Feng S. (2018). Diazotization-Coupling Reaction-Based Determination of Tyrosine in Urine Using Ag Nanocubes by Surface-Enhanced Raman Spectroscopy. Nanomaterials.

[26] Luca F., De L.B., Vincenzo M., Elena S., Renato M., Maria G.B., Danila M., Fabiana A. (2021). Smartphone-assisted electrochemical sensor for reliable detection of tyrosine in serum. Talanta.

[27] Chen H.Y., Yeh Y.C. (2020). Detection of tyrosine and monitoring tyrosinase activity using an enzyme cascade-triggered colorimetric reaction. RSC Adv..

[28] Lin C., Jair Y.C., Chou Y.C., Chen P.S., Yeh Y.C. (2018). Transcription Factor-Based Biosensor for Detection of Phenylalanine and Tyrosine in Urine for Diagnosis of Phenylketonuria. Anal. Chim. Acta.

[29] Feng D.Q., Liu G., Ma G., Nan Z., Wang W. (2018). Phosphodiesters quaternary ammonium nanoparticles as label-free light scattering probe for turn-off detection of tyrosine. Spectrochim. Acta Part A Mol. Biomol. Spectrosc..

[30] Huang G.G., Yang J. (2005). Development of infrared optical sensor for selective detection of tyrosine in biological fluids. Biosens. Bioelectron..

[31] Huan W., Hui Z., Dagan Z., Jie W., Hui T., Tiantian K. (2021). Enzyme-functionalized structural color hydrogel particles for urea detection and elimination. J. Clean. Prod..

[32] Rinky S., Kikuo K., Sushmee B. (2017). Graphene–Polyaniline composite based ultra-sensitive electrochemical sensor for non-enzymatic detection of urea. Electrochim. Acta.

[33] Lu S., Gu Z., Hummel M., Zhou Y., Wang K., Xu B.B., Wang Y., Li Y., Qi X., Liu X. (2020). Nickel Oxide Immobilized on the Carbonized Eggshell Membrane for Electrochemical Detection of Urea. J. Electrochem. Soc..

[34] Liu J., Moakhar R.S., Perumal A.S., Roman H.N., Mahshid S., Wachsmann-Hogiu S. (2020). An AgNP-deposited commercial electrochemistry test strip as a platform for urea detection. Sci. Rep..

[35] Liu Q., Stenbæk Schmidt M., Thienpont H., Ottevaere H. (2020). A Tunable Freeform-Segmented Reflector in a Microfluidic System for Conventional and Surface-Enhanced Raman Spectroscopy. Sensors.

